# Plant cuttings of invasive alien *Impatiens glandulifera* Royle develop flowers and produce viable seeds

**DOI:** 10.1038/s41598-025-33573-8

**Published:** 2026-02-17

**Authors:** Kamil Najberek, Monika Myśliwy, Agnieszka Rewicz, Wojciech Solarz

**Affiliations:** 1 Institute of Nature Conservation, Polish Academy of Sciences, Al. Adama Mickiewicza 33, Kraków, 31-120 Poland; 2 Institute of Marine and Environmental Sciences, University of Szczecin, Adama Mickiewicza 16, Szczecin, 70-383 Poland; 3 Department of Geobotany and Plant Ecology, University of Lodz, Banacha 12/16, Łódź, 90-237 Poland

**Keywords:** Biological invasions, Cost-effectiveness, Invasive alien species (IAS), Management, Nonlinear environmental effects, Survival mechanisms, Plant reproduction, Flowering, Pollination, Seed development, Invasive species

## Abstract

Controlling the invasive alien Himalayan balsam *Impatiens glandulifera* is both expensive and time-consuming. The most promising control methods include manual mowing and hand-pulling. However, long-term effectiveness of these methods can be unsatisfactory. We hypothesized that such partial success might result from eradication efforts occurring too late in the season, allowing improperly treated cuttings to flower. Moreover, we experimentally tested whether cut flowering individuals left on the ground could survive long enough to produce viable seeds. The experiment involved plant cuttings and reference individuals. We recorded the number of flowers on each plant, monitored floral visitor activity, and simultaneously measured abiotic factors (air temperature, solar radiation, wind speed). The relationship between insect visitation rates and both flower abundance and solar radiation was nonlinear, highlighting the importance of considering complex environmental effects in pollination dynamics. The seeds were collected, and seed viability was compared between the two treatment groups. We demonstrated that the plant cuttings survived for about three weeks, developed flowers and produced viable seeds. Although the reference individuals had more flowers and were more frequently visited by insects (mainly *Bombus pascuorum*), they did not develop significantly more viable seeds than the cuttings. This confirms the remarkable survival capacity of *I. glandulifera* plant cuttings and supports our hypothesis that this feature may hinder the full eradication of the species through mowing and hand-pulling under current guidelines. Based on our results, we recommend changing the control timing.

**Supplementary Information:**

The online version contains supplementary material available at 10.1038/s41598-025-33573-8.

## Introduction

 The Himalayan balsam (*Impatiens glandulifera* Royle) is an annual invasive alien plant native to the western Himalayas. It was first introduced to Europe in the mid-19th century, initially cultivated at Kew Gardens in the United Kingdom, from where it subsequently escaped into the wild^1,2^. The species underwent a lag phase before becoming invasive, and its rapid spread has been documented since the late 20th century^3^. Currently, its invasive range covers most European countries and extends into transcontinental regions between Europe and Asia – the Balkans and the Caucasus. In Asia, its new localities have been recorded in the Russian Altai Republic, China’s Hunan Province, and Japan. In addition, the species has also been introduced to North America and Argentina^4^.

The primary reason for the frequent intentional introductions of *I. glandulifera* was its ornamental value and high nectar production. Cultivation in gardens and its promotion by beekeepers further facilitated the species spread, as its flowers provide extraordinary nectar rewards to pollinators^5^. Notably, the sugar production rate of *I. glandulifera* flowers (0.47±0.12 mg h^–1^ per flower) is much higher than that of most other European wild-growing plants^6^. Moreover, the nectar of this alien species contains more chemical attractants than that of its native congener, *I. noli-tangere*^7^. Unintentional introductions occur through disposal of garden waste, both legal and illegal, often near water bodies – the preferred habitat of *I. glandulifera*^8^. After establishment, the species spreads naturally through ballistic seed dispersal (up to five meters), as well as by water, or through animals carrying seeds on their fur or feathers, or via ingestion. Human-mediated dispersal also occurs through contaminated soil or waste, during river engineering works, and unintentional transport with crops or vehicles on land and water^9^.

Nowadays, *I. glandulifera* is among the most widespread alien plant species in Europe, exerting a significant negative impact on native biological diversity. This success is related to its high phenotypic plasticity^10^ and/or evolutionary shifts that the species has undergone following introduction^11^. In invaded areas, the species often forms dense, tall stands that suppress the growth of co-occurring native plants, leading to a shift in floristic composition toward shade-tolerant and nitrophilous species^12,13^. In contrast, in its native range *I. glandulifera* individuals are much smaller and more scattered, and do not form dense monocultures^14^. It is known that extensive patches of the species may reduce diversity of co-occurring native plants by 25%^15^. The species presence also significantly alters the habitat microclimate, affecting terrestrial animals (e.g., gastropods^16^.

In its European range, the reproductive potential of *I. glandulifera* (i.e. the development of flowers, capsules and seeds) is higher compared to native populations^17^, as is plant density per square meter^17^. This could be a result of the species’ high potential to release from its natural enemies^17^, which was confirmed by biogeographical comparisons between native and introduced populations^18^. However, some studies have reported contrasting results^19^. What is unequivocal, however, is *I. glandulifera*’s extraordinary potential to lure pollinators away from co-occurring wild native species^6^ and crops^20,21^, which poses a serious threat to native biodiversity and agricultural productivity, and further facilitates the invasion of this impactful alien species. In addition, invasion of *I. glandulifera* has adverse economic consequences, as its flowers enhance the transmission of harmful pathogens that cause severe diseases in both crops and native plants (e.g., *Botrytis cinerea*^22^.

Controlling the invasion of *I. glandulifera* is challenging both in terms of efficiency and costs, confirming that the knowledge about management methods of this plant species is limited^23^. The most promising means of control include repeated mowing and hand-pulling^24–29^. Mowing is applied in large populations or at steep banks of rivers and lakes, when hand-pulling could disturb soil cohesion and lead to water erosion; in turn, hand-pulling is recommended for controlling small patches of the species, as well as in areas where it coexists with valuable native species and has invaded protected natural habitats where mowing is not advisable. Manual control may cost as much as £0.5/m^2^^30^.

These two methods are complementary, however, even if repeated for several subsequent years, the long-term control effects can be unsatisfactory. For example, ten years of intensive mowing and hand-pulling in the Thayatal-Podyjí National Park reduced the invaded area by 77%, yet surviving individuals still posed a threat^28^. Thus, it is concerning whether complete eradication is possible to achieve, taking into account that expensive and time-consuming control is conducted as long as financing is available.

Therefore, improving methods to control this harmful species is particularly important in light of its further spread in Europe – not only along river valleys and in disturbed habitats, but also into forests^31^. Studies on the biology of the Himalayan balsam seeds have revealed their high germination potential, even in immature seeds^32,33^. The species can persist in the soil seed bank for up to four years. In comparison, another invasive alien balsam, *I. parviflora*, germinates only in the first year after seed burial, while the native *I. noli-tangere* may germinate as late as in the fifth year^34^. These findings demonstrate the remarkable survival capacity of *I. glandulifera* and provide valuable insights for developing more effective management and control strategies for this highly invasive alien species.

Previous studies and field observations have shown that mechanical control of *I. glandulifera* can be ineffective when applied too late or performed improperly^23,26,29,35,36^. Clipped stems are capable of developing flowering shoots if cut too soon^22,25,33^, while pulled plants can readily root from nodes^25^, allowing regeneration and subsequent seed production. These regenerative traits significantly reduce the efficiency of mowing and hand-pulling, emphasizing the need for careful timing and proper disposal of removed plant material.

Interestingly, recent field observations in southern Poland have revealed a noticeable advancement in the flowering period of *I. glandulifera*. In the vicinity of Kraków, the species started flowering as early as in the first half of July in 2022 and 2023, whereas in 2024, in the mountain town of Zakopane, the first blooming flowers appeared already on 20 June (Rewicz, Myśliwy, Najberek, pers. observ.). These findings suggest that the phenology of *I. glandulifera* is shifting towards earlier flowering, likely in response to ongoing climatic changes. Such advancement in flowering time may have important implications for the efficiency and timing of control measures against this invasive alien species.

In the presented study, we investigated potential factors that lower the chance of full eradication of *I. glandulifera* by repeated mowing and hand-pulling. We assumed that the lack of success could be due to control measures applied too late in the season – at the beginning of flowering time – and to the improper disposal of plant material. We hypothesised that *I. glandulifera* plant cuttings with flowers or with flower buds may survive long enough to successfully produce viable seeds. To test this, we conducted a common garden experiment using *I. glandulifera* individuals transplanted from multiple field locations. Half of the plants were cut and left on the ground, while the rest served as a watered reference group. We tracked environmental factors such as air temperature, wind speed, and solar radiation, monitored the activity of flower visitors, and assessed seed production and viability using a tetrazolium test. This approach allowed us to evaluate whether the timing and method of mechanical control can influence the reproductive success of the species and potentially explain its persistence despite eradication efforts.

## Methods

### Materials for research

*Impatiens glandulifera* is listed in the EU Regulation No 1143/2014 (2014)^37^  on the prevention and management of the introduction and spread of invasive alien species. Since our study involved wild-growing plants of this species, we were required to obtain permission from the Regional Directorate for Environmental Protection (No.: OP.672.2.2021.KW, OP-1.640). As stipulated in the conditions of this permission, all plants and seeds had to be destroyed after the completion of the tests to prevent any risk of dispersal. As a result, no voucher specimens of plant material were deposited in a publicly accessible herbarium. Additionally, we obtained the necessary permit (OP-1.640 1.81.2021.GZ) to study legally protected bumblebees visiting the flowers.

The experiment was conducted in 2021 under common garden conditions in a cultivation plot localised in Kraków, in southern Poland (50.089741 N, 19.864134 E). In May 2021, seedlings of *I. glandulifera* (*N* = 40) were transplanted to the plot from the following localities: Marcyporęba (49.922791 N, 19.626132 E), Ochodza (49.976944 N, 19.749985 E), Tyniec (50.020002 N, 19.819796 E), and Szczyglice (50.086645 N, 19.814899 E). Although no major differences were expected under controlled conditions, individuals were collected from multiple localities to account for potential site-related variation and increase the generality of the findings. Each locality, represented by 10 seedlings, was located within a radius of 20 km from the plot. The species identification was performed by the first author. The plants were cultivated in pots (1.1 L capacity) filled with universal garden soil mixed with sand in a 3:1 ratio. Each plant was tagged with the individual ID.

**Fig. 1:**
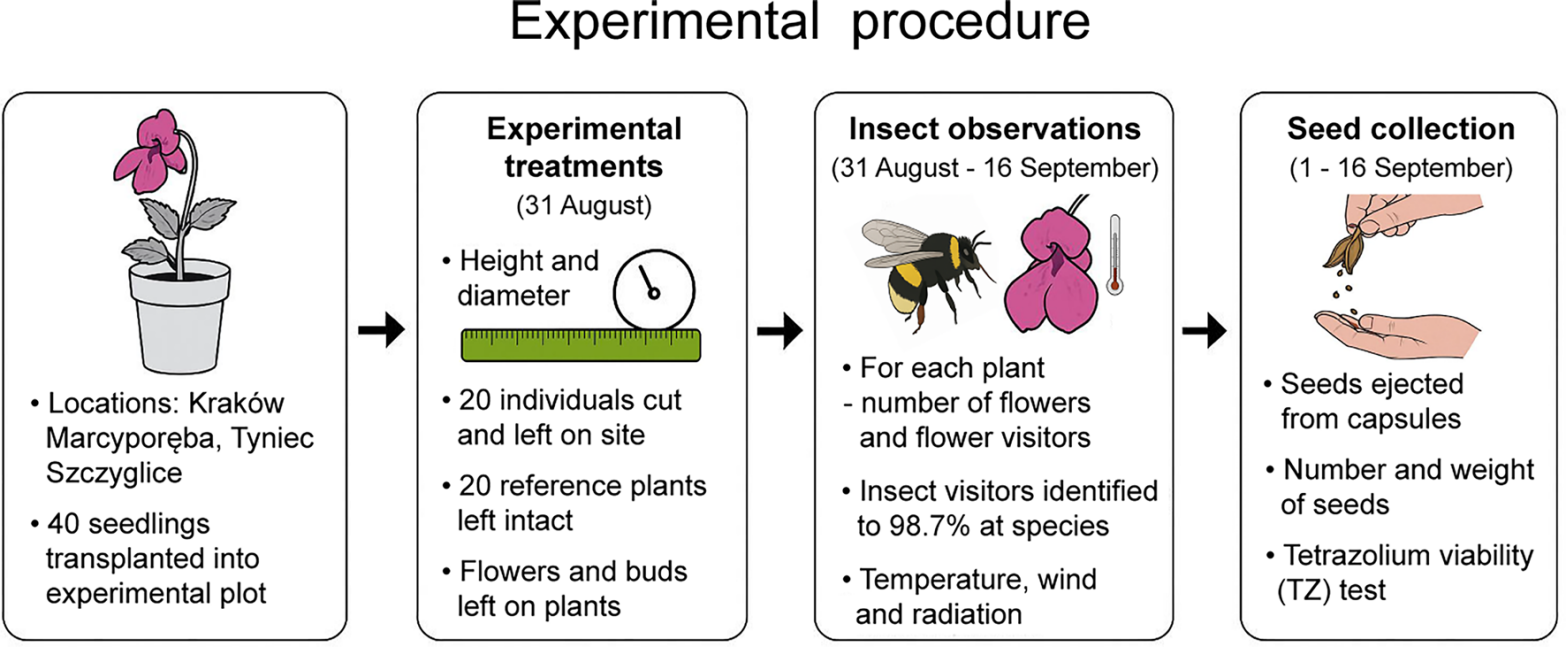
Overview of the experimental design involving *Impatiens glandulifera* seedlings, pollinator observations, and seed viability testing. Figure was generated with assistance from OpenAI’s ChatGPT, using a text-to-image model. The initial output was subsequently edited and refined by the authors.

 Because taller and larger plants may attract more floral visitors (taller stems may make flowers more detectable to pollinators^21^, and larger individuals may offer more floral resources^38^, on August 31 we measured stem height (from the ground to the apex) and stem diameter (near the ground) of each flowering individual (Table S1). At the time of cutting, all individuals were at the mid-flowering stage, typically bearing both open flowers and flower buds, while early-stage fruit capsules were also present on the tested plants. All developed seed capsules, flowers and flower buds were left. Subsequently, 20 individuals (5 per locality; hereinafter ‘plant cuttings’) were cut off from the root near the ground and placed on the ground. The remaining 20 individuals were used as a reference sample. In contrast to the plant cuttings, the reference individuals were still watered. See Fig. 1 for an overview of the experimental design.

To avoid positional bias, the assignment of individuals to the two treatment groups was conducted randomly with respect to source location. In addition, randomized positioning of individuals was applied within each treatment group. The groups themselves, however, were not spatially mixed, because the aim of the experiment was to compare the species’ potential under two distinct conditions: (1) reference individuals (before eradication) and (2) plant cuttings (after eradication). For this reason, the cuttings were placed on the ground directly adjacent to the reference individuals, ensuring comparable environmental conditions across the groups. We acknowledge that certain factors inevitably differed between treatments (e.g., wind speed being lower near the ground), but these differences were intrinsic to the biological scenario we intended to mimic and were thus an integral part of the experimental design. Therefore, it was particularly important to include meteorological data in the statistical analyses to account for such environmental variation.

## Assessment of flower visitor activity

The experiment was carried out for 17 days in the flowering period of *I. glandulifera* (mid-flowering: August/September 2021^22^. The first five surveys were carried out over five consecutive days (dates: August 31 to September 4), immediately following the cutting of plants, on August 31, while the last two – five and seven days later (September 9, 16). Each study day, the number of flowers on each plant cutting and reference individual were counted, followed by tallying the number of flower visitors in both groups (Fig. 1). We did not assess if a given visit actually resulted in pollination, therefore we conservatively do not treat the floral visitors as pollinators. However, it can be assumed that the majority of the visiting insects that we recorded were in fact pollinators.

Each survey was carried out by the same researcher and with the same sampling effort. The counting of flower visitors commenced between 11:00 and 15:00 and was carried out through a 60-minute period. During each survey, the flight of each recorded flower visitor was tracked, and ID numbers of subsequently visited balsam flowers of the plant cuttings and reference individuals were noted. The insects were identified at flowers (98.7% to the species level, 1.1% to the family and 0.2% to the genus), without catching.

Weather conditions differed between the treatment groups (plant cuttings were placed on the ground). However, since this is another factor that may influence activity of insects^21^, air temperature, wind speed and solar radiation were measured during the surveys (Fig. 1). The temperature was measured using data loggers (i-Button DS1921G) with 5-min intervals. Wind speed and solar radiation were measured using environmental metres (Extech 45170CM and SP505, respectively) with 15-min intervals.

## Seeds and their viability

The developed seed capsules were successively wrapped with plastic bags (dimensions: 40 × 60 mm) to collect all ballistically projected mature seeds. However, the capsules were not wrapped immediately after development; instead, they were allowed time to grow. They were wrapped around the midpoint of the maturation cycle, based on visual assessment. The seeds projected to the bags were collected and then used to compare seed viability of the plant cuttings with the reference individuals (Fig. 1). The seeds were collected between September 1 and 16, counted and weighted using an analytical balance (Radwag PS 360.R2). Their viability was assessed with the use of the tetrazolium (TZ) test, following the methods adapted for *I. glandulifera* in our previous studies^8,39^; the seed embryos stained red using 1% tetrazolium solution (pH ± 7) were categorized as viable.

### Statistical analysis

The data were analyzed using R v. 4.4.3 and RStudio v. 2024.12.1 + 563. Flower visitor activity in the plant cuttings and reference individuals was tested using generalized additive models GAMs. The models were fitted using the mgcv package^40^, with thin plate regression splines^41^, restricted maximum likelihood estimation (REML^42^, and automatic smoothing parameter selection^43^. The number of the recorded insect visits (N visits), calculated per plant individual per survey, was included as a response variable in the models (*n* = 709).

 Initially, two GAMs were compared to assess the presence of zero inflation in the count data: (1) a zero-inflated Poisson GAM and (2) a standard Poisson GAM. Both models included treatment group (plant cuttings vs. reference individuals) as a parametric factor and smooth terms for the number of flowers, air temperature (ºC), wind speed (m/s), solar radiation (wm^2^), stem height (cm), stem diameter (mm); and random effects for plant individual ID (Plant ID) and the number of days since the start of the experiment (Study day), both modelled as smooth terms with a random effect basis (bs = “re”). For the continuous predictors (number of flowers, air temperature, solar radiation, wind speed), smooth terms were fitted separately for each treatment group using the “by” parameter (e.g., s(N_flowers, by = Treatment_group)) to allow different smooth relationships between the predictors and pollinator visits across groups. Model comparison based on Akaike Information Criterion (AIC) showed that the zero-inflated Poisson GAM (AIC = 1467.9) fitted the data better than the Poisson GAM (AIC = 1969.5), indicating significant zero inflation in the response variable; moreover, the Poisson GAM exhibited clear violations of model assumptions (Figure S1). Subsequently, the zero-inflated Poisson GAM was simplified using the argument select = TRUE to perform automatic variable selection and smoothing parameter estimation. The resulting reduced model included treatment group, number of flowers, solar radiation, wind speed (all with group-specific smooths), and random effects for plant ID and study day. Although the reduced model had a marginally higher AIC (AIC = 1470.1) compared to the full zero-inflated model (AIC = 1467.9), it provided a clearer and more interpretable representation of the data and was therefore selected for the final inference. Model diagnostics were performed using the gam.check function and residual simulations from the DHARMa package^44^ to assess model fit and assumptions. These checks indicated satisfactory model convergence and no violations of distributional assumptions. Summary statistics and diagnostics for all models are provided in the supplementary materials (Figure S1, Tables S2 and S3).

The random effect plant ID was included to account for repeated measurements taken on the same plant individuals across different study days. This structure allowed us to control for plant-specific variability in floral attractiveness to visitors, which could not be fully explained by the fixed covariates such as stem height or diameter. Additionally, study day was included as a random effect to account for unmeasured day-specific sources of variation, including both fluctuating abiotic factors not fully captured by the fixed predictors (air temperature, solar radiation, wind speed), as well as progressive changes in the physiological condition of the plant cuttings resulting from detachment from the root system. While some of the predictors included in the GAMs (such as weather conditions and morphological traits) are not directly part of the tested hypothesis, they were retained in the base model to control for known covariates that can influence flower visitation rates independently of the treatment effect. This approach allows for more accurate estimation of the treatment effect and strengthens the inference regarding our central hypothesis that *I. glandulifera* plant cuttings with flowers or flower buds can survive long enough to successfully produce viable seeds. This structure ensures that differences in pollinator visitation between treatment groups are not confounded by microenvironmental or temporal variation, allowing for a more accurate estimation of the treatment effect, particularly given that cut stems and reference plants experienced systematically different exposure to environmental conditions.

Differences in the number of viable seeds were tested using the proportion test (*n* = 1257). In turn, the average weight of a single seed, calculated for seeds from particular capsules (response variable), was analyzed using a linear model (*n* = 223). The explanatory variable was the treatment group (plant cuttings/reference individuals). The interaction between the treatment group and study day was also included in the model. The interaction allowed to compare shifts in seed weights over time between the plant cuttings and reference plants.

## Results

### Flower production

**Fig. 2:**
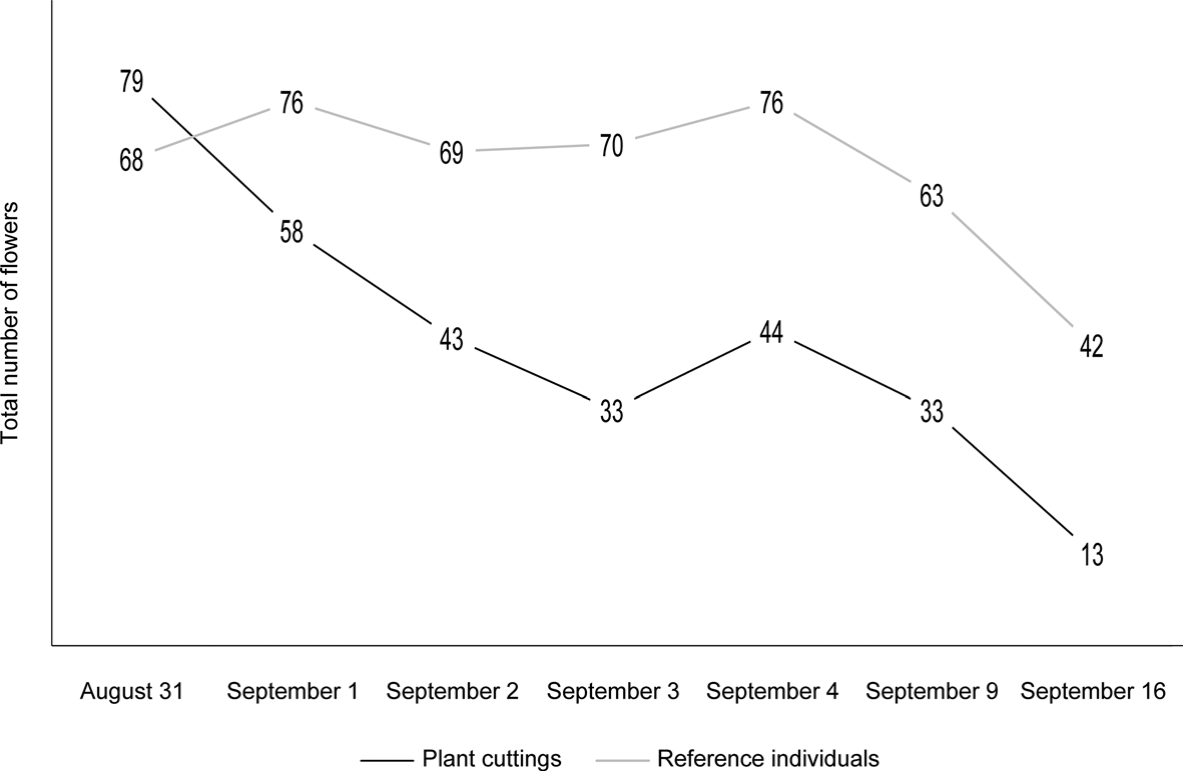
The number of flowers of all individuals of *Impatiens glandulifera* per treatment group and study day.

The insects were recorded from an average of 55 flowers regardless of the treatment group. This number decreased over time, which was particularly evident among the plant cuttings (Table 1). Although the total number of flowers was initially higher in the plant cuttings, at the end of the experiment it was twice as low (Table 1; Fig. 2). However, it should be stressed that both the plant cuttings and reference plants developed new flowers during the entire experiment (accounting for 12.5% and 13.8% of all flowers, respectively); e.g., the plant cutting individual with ID 17 developed one new flower between September 2 and 3, another between September 3 and 4, and six new flowers between September 9 and 16 (a total of 8 newly developed flowers; Table 1). There were only two plant cutting individuals that did not develop any new formed flowers (plants with IDs 24 and 37; Table 1). In general, the plant cuttings developed half as many flowers as the reference individuals (Table 1; Fig. 2).

**Table 1:**
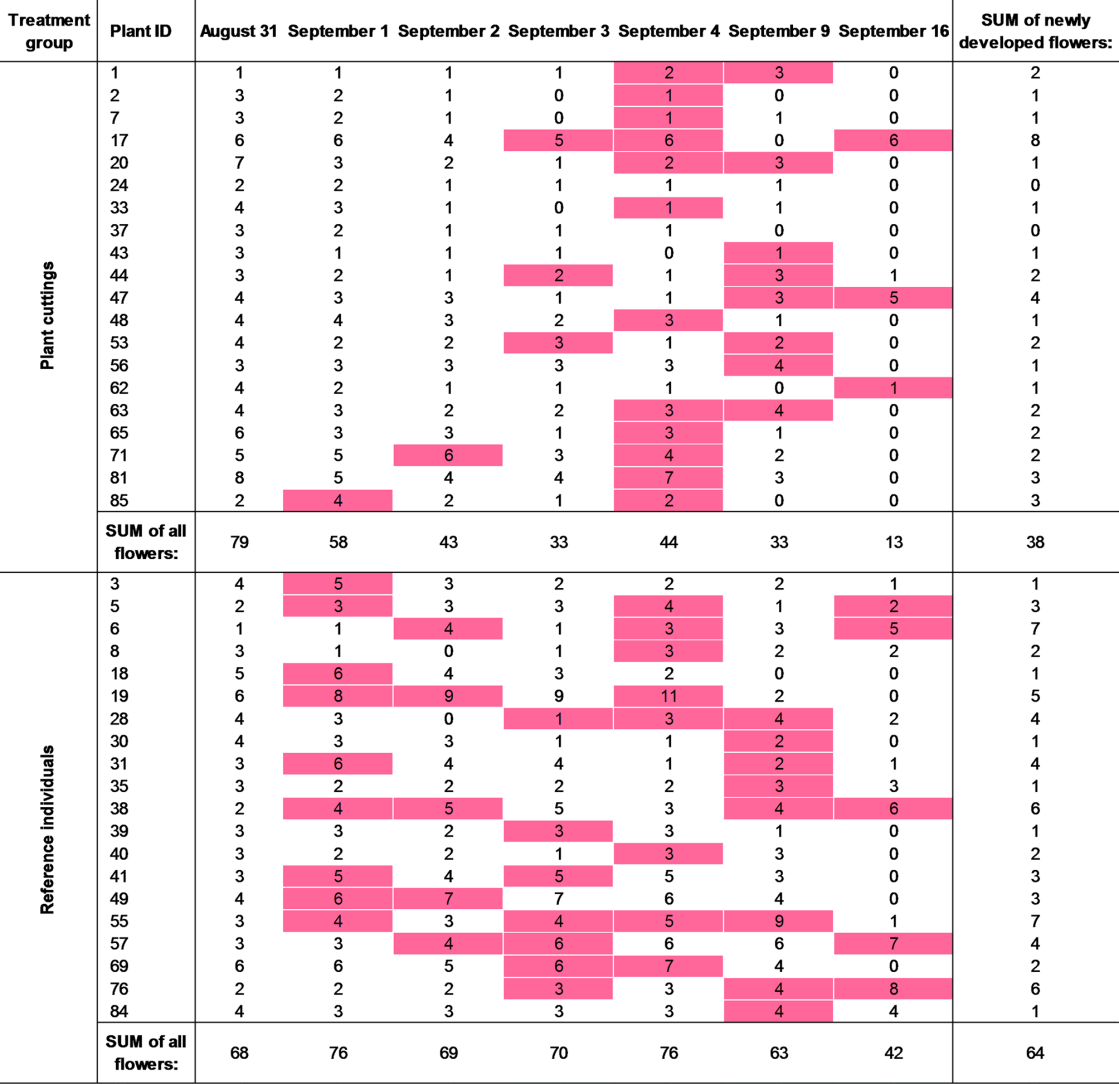
The number of flowers available for pollinators on each plant individual in consecutive study days, in both treatment groups. Days in which new flowers were detected are marked in pink and the number of new flowers is summarised in the last column. E.g. Plant ID 17 developed a total of 8 new flowers, including 1 between Sep 2/3, 1 between Sep 3/4, and 6 between Sep 9/16. Detailed information on the number of flowers per survey and treatment group is provided in supplementary table S4.

## Assessment of flower visitors activity

In total, 144 insect flights resulting in 1195 insect visits to the flowers were recorded. Most of the visits were recorded from the reference plants (94.6%), whereas the flowers of the plant cuttings were visited much less frequently (3.4%). In both treatment groups common carder bee *Bombus pascuorum* was the dominant visitor (Table 2).

**Table 2:**
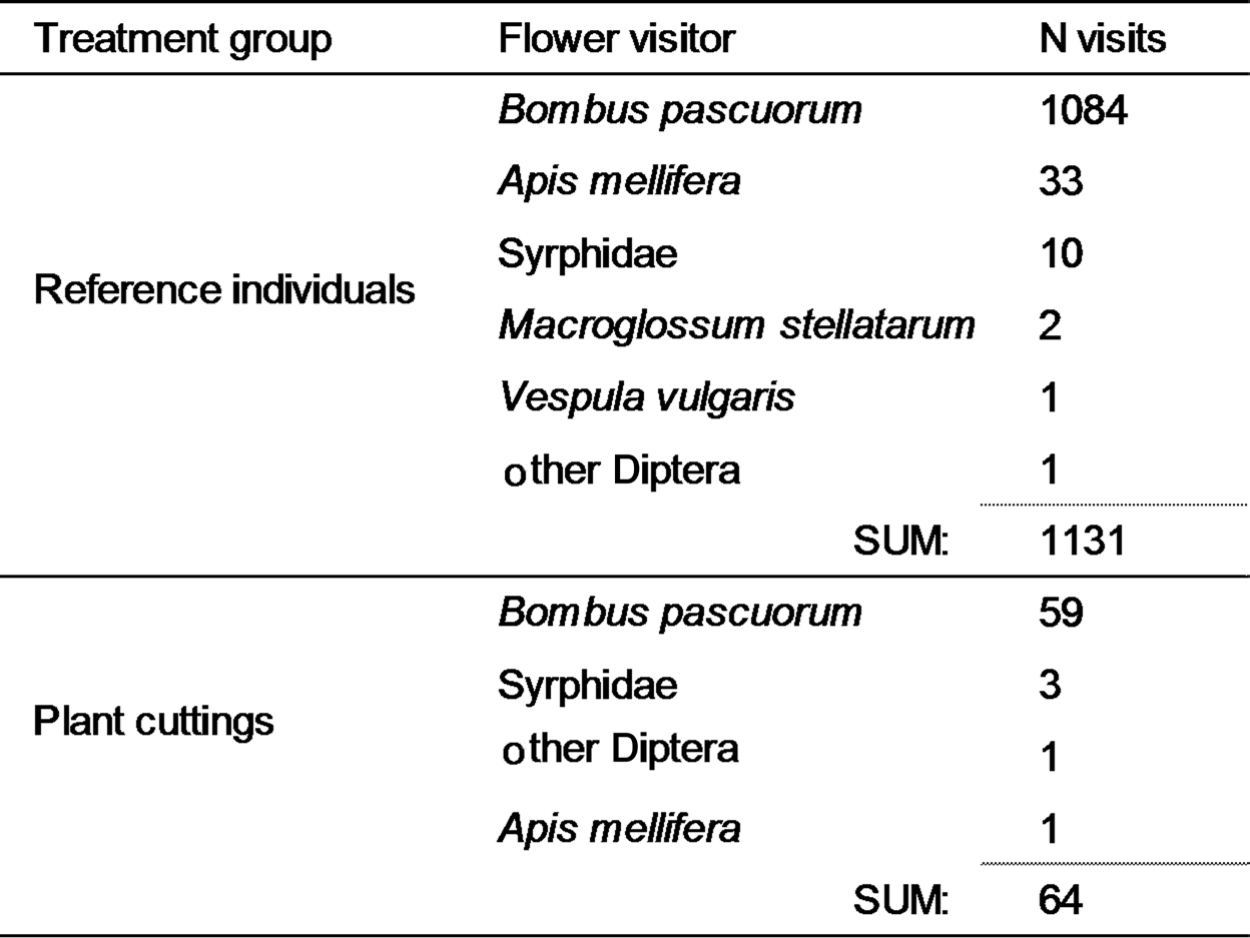
The number of insect visits to the flowers of *Impatiens glandulifera* per treatment group.

**Fig. 3:**
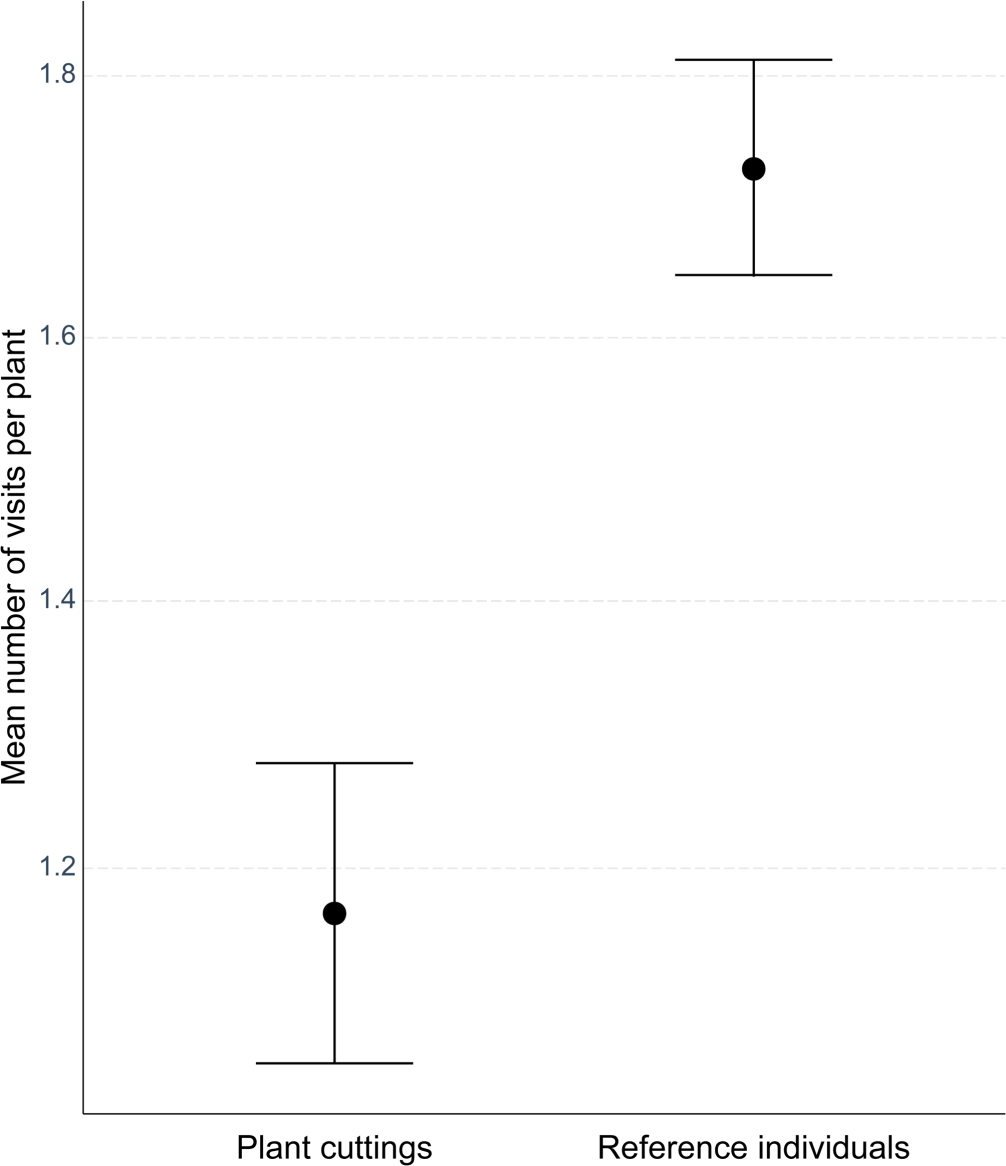
The mean number of insect visits (± SE) to the flowers calculated per each plant cutting and reference individual of *Impatiens glandulifera.*

**Fig. 4:**
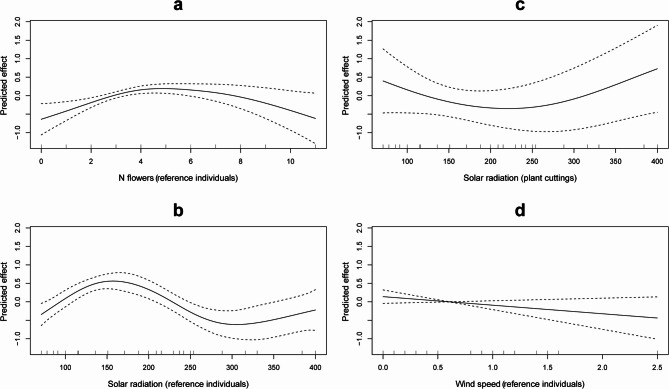
Estimated partial effects (smooth functions) of predictor variables on insect visit counts from a GAM with a zero‑inflated Poisson distribution. Panels show the smoothed effects with 95% confidence intervals (solid and dashed lines) for: (**a**) number of flowers, (**b**,** c**) solar radiation (wm^2^) and (**d**) wind speed (m/s). The y‑axis represents the contribution of each predictor (smooth term) to the linear predictor scale (link function) after accounting for other variables in the model.

A generalized additive model (GAM) with a zero-inflated Poisson distribution was used to assess the effects of treatment group, number of flowers, solar radiation and wind speed on the number of insect visits (Table 3; see also Figure S1, Tables S2 and S3). The model accounted for excess zeros in the data and included random effects for plant ID and study day to control for repeated measures and temporal variation. The model explained 28.9% of the deviance in pollinator visitation rates (REML = 743.88, *n* = 709). The number of insect visits in flowers of the reference individuals was significantly higher than in the plant cuttings (*p* < 0.001; Fig. 3; Table 3). Smooth terms revealed a significant, nonlinear relationship between the number of flowers and pollinator visits in the reference individuals (*p* = 0.002; Table 3), characterized by an initial positive trend, followed by saturation at higher flower numbers (Fig. 4a). In contrast, no such relationship was found in the plant cuttings (*p* = 0.98; Table 3). Similarly, solar radiation showed a strong nonlinear effect on pollinator visits in the reference individuals (*p* = 0.0017; Fig. 4b; Table 3), but only a marginal effect in the plant cuttings (*p* = 0.096; Fig. 4c; Table 3). Higher solar radiation was associated with increased pollinator activity, although the response appeared to decline at very high radiation levels. Wind speed had no significant effect in the plant cuttings (*p* = 0.94), and a marginally significant, negative linear effect in the reference individuals (*p* = 0.053; Fig. 4d; Table 3). Random effects for plant ID (*p* = 0.028) and study day (*p* < 0.001) were significant, indicating substantial variability among individual plants and across study days (Figures S2a and S2b; Table 3).

**Table 3:**
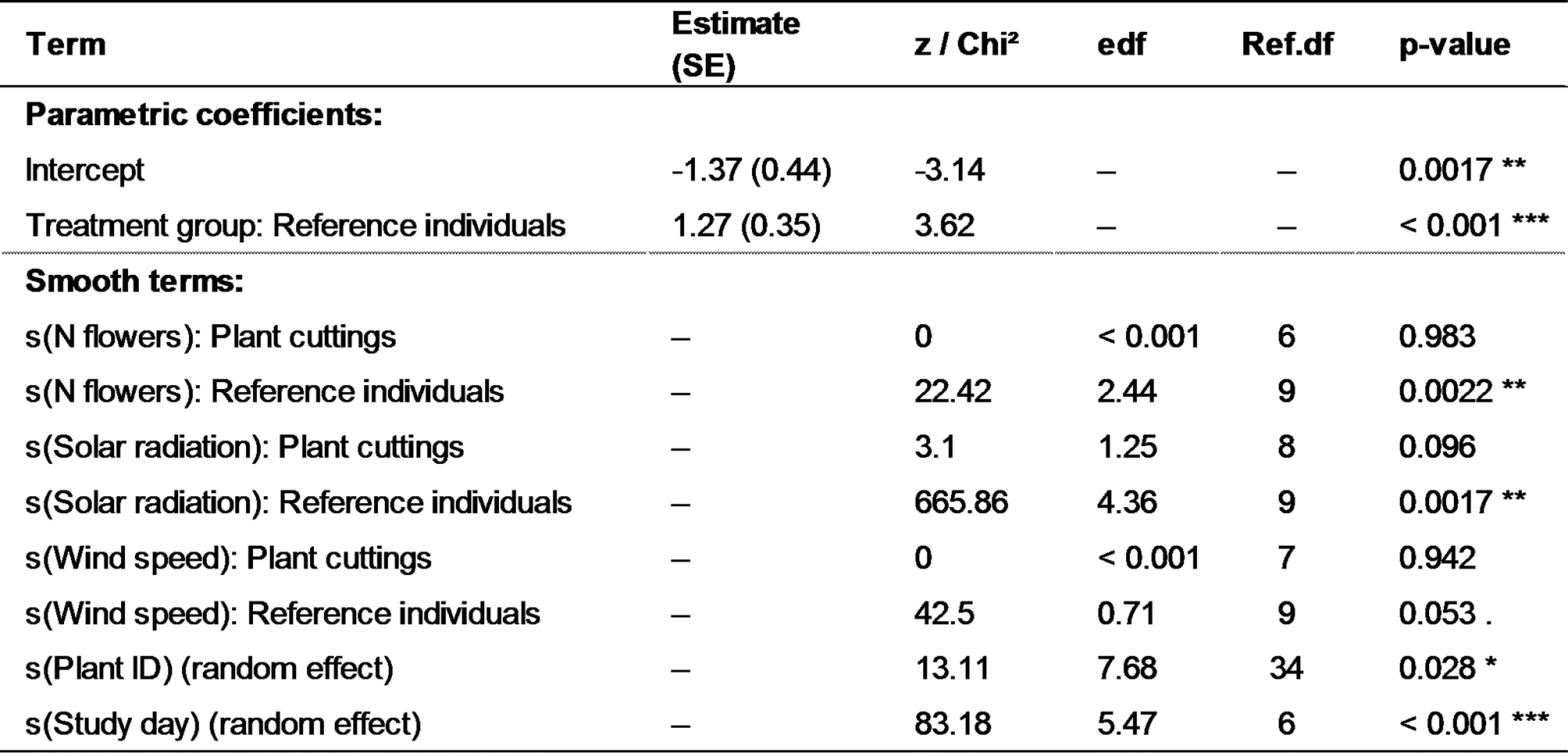
Results of the generalized additive model (GAM) for the number of insect visits. Values are shown as parameter estimates ± standard error (SE) for fixed effects and as Chi² statistics for smooth terms. edf = estimated degrees of freedom; Ref.df = reference degrees of freedom. Significance levels: * *p* < 0.05, ** *p* < 0.01, *** *p* < 0.001, *p* < 0.1. Model: zero-inflated Poisson family with REML estimation, deviance explained = 28.9% (see also Figure S1, Tables S2 and S3).

## Seeds and their viability

In total, 1257 developed seeds were collected: 592 from the plant cuttings and 665 from the reference individuals. In the former group, 159 of the developed seeds were viable (27%). In turn, the reference individuals developed 149 viable seeds (22%); this difference was not statistically significant (χ2 = 3.35, *p* = 0.07).

**Fig. 5:**
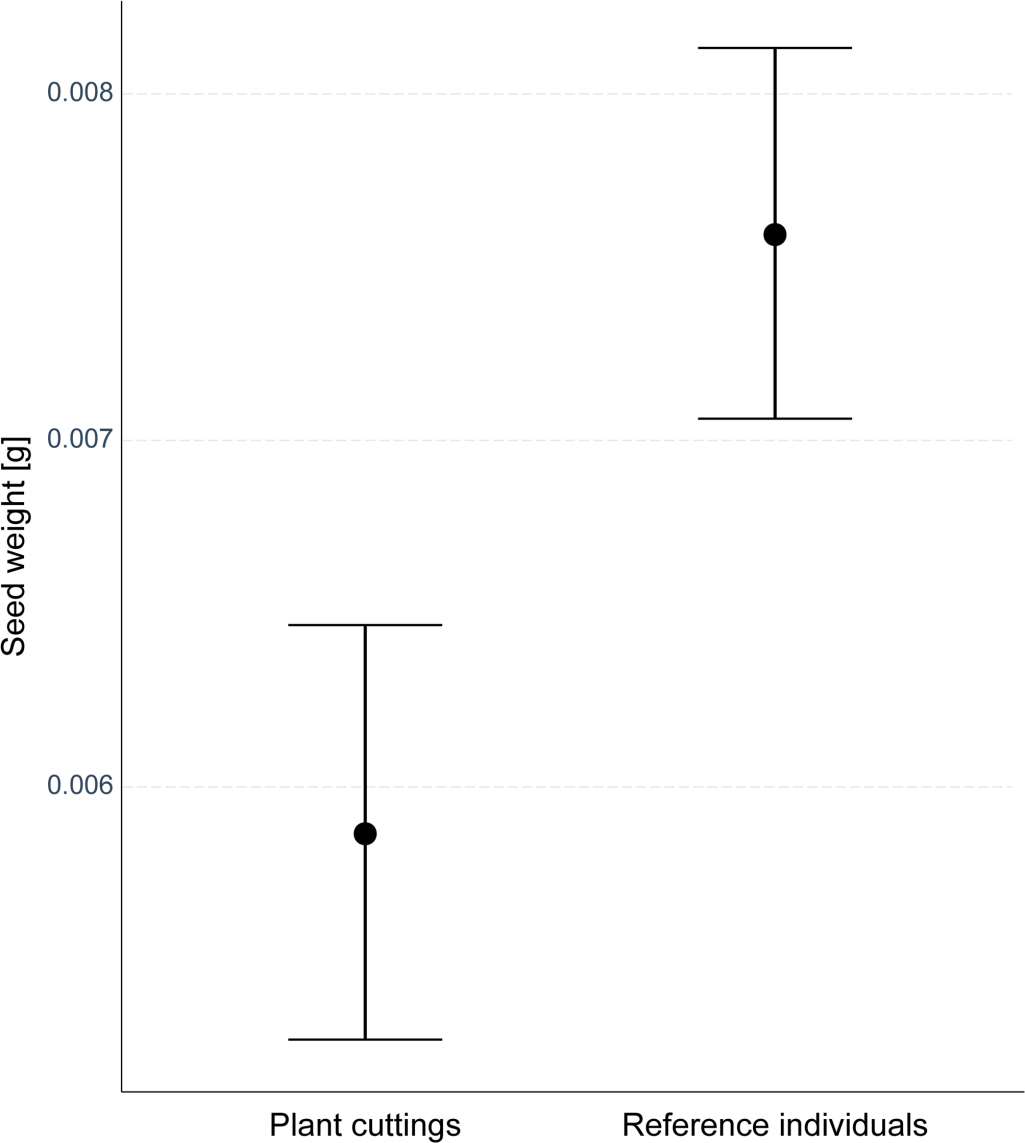
The estimated mean weight of seeds (± 95% confidence intervals) collected from the plant cuttings and reference individuals of *Impatiens glandulifera*.

The average weight of a single seed collected from the plant cuttings was 0.0058 ± 0.0025 g. The seeds of the reference plants were heavier and weighted on average 0.0076 ± 0.0034 g. The revealed difference in seed weight between the groups was statistically significant (Sum Sq = 0.00017, F_(1, 222)_ = 18.58, *p* < 0.001; Fig. 5). In turn, the interaction between the treatment group and study day did not influence the results (Sum Sq = 0.000015, F_(1, 222)_ = 1.68, *p* = 0.2), therefore the weight of seeds did not change along with successive experiment days.

## Discussion

With the EU Regulation No 1143/2014 (2014), the prevention against the introductions and spread of invasive alien species is legally supported at the European Union level. The provisions of this Regulation were also implemented in Member States^45,46^. Due to these legal developments the level of recognition of biological invasions as a serious threat to biodiversity and economy has increased^47,48^. Nevertheless, despite the expensive and time-consuming management of invasive alien species, the effects are often not satisfactory. For example, it is known that eradication of *Impatiens glandulifera* may not be feasible^28,49^.

The successful eradication of *I. glandulifera* clearly depends on the extent of its dispersal within the control site. However, our results indicate that optimizing eradication procedures can substantially enhance their effectiveness. We suggest that survival of this species in eradication areas could be driven primarily by two factors: control applied too late in the season, and improper disposal of plant material produced during control practices. We found that *I. glandulifera* can survive for approximately three weeks after being severed from its roots. During this period the tested plant cuttings were able to develop new flowers that were additionally visited by insects. Importantly, the number of newly produced flowers was only half as many as in the healthy reference plants. Nevertheless, flower initiation continued throughout the experiment in both groups (12.5% and 13.8% of all flowers in plant cuttings and in reference plants, respectively). For instance, one individual produced eight new flowers after cutting, while only two individuals failed to develop any new ones. This indicates that reproductive activity was not completely suppressed by the treatment. Importantly, both pre-existing and newly developed flowers produced viable seeds, confirming that plant cuttings can complete seed maturation even from partially developed reproductive organs. Under natural conditions, this capacity may enable the species to persist within managed sites.

The role of abiotic factors in pollination should also be emphasized. The non-linear smooth terms identified in our GAM analysis revealed that the effects of environmental variables on pollinator visitation were context-dependent and differed significantly between the treatment groups. In the reference individuals, both flower abundance and solar radiation exhibited strong non-linear relationships with visitation rates, indicating that flower visitors’ responses are not directly proportional to these variables. For example, in the reference individuals, the number of visits increased with flower number and solar radiation up to certain thresholds, beyond which the effects plateaued or slightly declined. In contrast, no significant smooth effects were observed in the plant cuttings, suggesting that their overall lower attractiveness might limit pollinator sensitivity to environmental changes—that is, both to direct abiotic factors such as solar radiation and to their indirect consequences, including variation in flower production. This pattern also indicates that pollinators did not respond to increasing flower numbers on cut stems in the same way as they did on intact plants, consistent with the idea that cuttings are less attractive. Consequently, the relationship between the two variables (flower abundance and solar radiation) and pollinator visitation is complex and cannot be characterized by a simple linear trend, highlighting the importance of considering non-linear environmental effects in pollination studies. At the same time, the linear relationship between wind speed and flower visitor activity showed a marginally significant positive effect on visitation rates in the reference individuals, while its effect was negligible in the plant cuttings. This difference may be explained by the fact that the plant cuttings were placed on the ground, likely reducing their exposure to wind and solar radiation compared to the reference plants. As a result, the influence of wind and solar radiation on insect visits was markedly weaker in the plant cuttings. These findings highlight the combined importance of plant quality and microenvironmental conditions in shaping plant–pollinator interactions under field conditions. Importantly, the inclusion of abiotic covariates such as solar radiation and wind speed, together with study day as a random effect, in our GAM, ensures that the observed differences between treatment groups were not confounded by microenvironmental or temporal variation. This approach allowed for a more accurate estimation of treatment effects on pollinator visitation, accounting for potential differences in exposure of cuttings and reference plants to environmental factors.

At the same time, we found that the weight of the seeds from the plant cuttings was lower than the weight of seeds collected from the reference individuals, which could suggest their lower performance. Lighter seeds from the plant cuttings could result from less intensive insect visitation than in the case of flowers of the reference plants^50^. However, after the experiment start date, only one-tenth of the newly developed seeds appeared in both treatment groups. This suggests that most flowers may have already had the opportunity to be pollinated prior to the experiment’s commencement. Therefore, it is likely that the reduced seed weight produced by the plant cuttings is associated with the lower performance of these plants. Nevertheless, the seeds collected from the plant cuttings, albeit in a worse condition, were as viable as the reference seeds, thus could be successfully dispersed within the controlled area.

According to the European Union risk assessment guidelines^26^, *I. glandulifera* should be manually removed before May or at the end of July, with at least two control treatments per season. The second treatment should be carried out at the beginning of the flowering period, before seeds are developed. Similar recommendations are given for eastern provinces of Canada^23,51^. However, flowering in Poland often begins earlier, sometimes as early as late June in the south (Rewicz, Myśliwy, Najberek, pers. observ.). Therefore, in light of our results and the ongoing shifts in species phenology likely driven by climate change, the currently recommended timing for the second treatment should be reconsidered. Implementing the control measures no later than early June would ensure greater effectiveness by entirely preventing flower development and seed production.

In Poland, the recommended management strategy for *I. glandulifera* includes five to seven control operations per year, with single post-control removals (“control removing”) permitted only when the population is at an early invasion stage^29^. The suggested timing of control application is always before the flowering phase, which is reasonable, but our results indicate that this rule should be specified more precisely. Plants removed only a few days before flowering were still able to survive long enough to produce flowers and viable seeds. Therefore, control measures should be implemented at least three weeks before the expected onset of flowering to prevent flower development and seed dispersal during the handling and destruction of plant material. This recommendation is based on our experimental observations showing that detached individuals of *I. glandulifera* remained turgid and capable of producing new flowers and viable seeds for approximately 17 days after cutting. The suggested three-week interval represents a conservative and safe threshold, ensuring that plants do not survive long enough to complete flower or seed development under field conditions.

It should also be noted that prior to flowering, *I. glandulifera* exhibits strong regenerative ability, which may reduce the efficacy of early control efforts^24,25,27,36^. However, rolling over cut stumps and removed plants could increase the effectiveness of eradication, as mechanical damage may inhibit the development of new stems and flower buds, thereby preventing subsequent fruit and seed production. Nevertheless, the efficiency of rolling over the plant material was not tested in the presented experiment and further studies are needed to confirm our supposition.

Proper disposal of plant material is another essential component of successful control management, given *I. glandulifera’s* high regenerative capacity. The EU risk-assessment^26,36^ provides only general recommendations and lacks specific operational guidance. In Canada, the recommended practice is to seal removed plants in black plastic bags and place them on impermeable surfaces for about one week, which effectively prevents regeneration^35^. In Poland, on-site disposal is advised, if possible, by covering with nets to prevent dispersal by wind or animals^29^. This method is practical for most invaded areas, although in protected sites the removed plants are usually collected, bagged, and burned elsewhere^27,52^. When permitted, on-site burning remains the safest and most cost-effective method, because transporting the plants to burn them in another location generates additional costs, requires more human effort and increases the risk of unintentional spread during the transport. Therefore, the bagging procedure^23^ appears to be a more realistic and cost-efficient option and should be considered the most adequate method for protected areas. Nevertheless, based on our findings, bagging plant cuttings for approximately three weeks (matching the maximum survival time of detached individuals) is sufficient to prevent seed development and should be considered a reliable method for safe disposal.

## Conclusions

The Himalayan balsam *Impatiens glandulifera* is an highly invasive alien plant species^1,2^. Although its eradication is expensive and time-consuming^30^, the complete removal of the species from invaded areas may be not feasible^28,49^. Mowing and hand-pulling are the two complementary methods that are recommended for the species removal. However, in light of our results, both methods should be revised to increase their efficiency. We found that plant cuttings of *I. glandulifera* may survive for approximately three weeks without roots, develop flowers visited by insects, and produce viable seeds capable of dispersal and germination, potentially leading to rapid re-invasion. To improve the efficiency of control methods, we therefore suggest adjusting their timing: based on our observations that detached individuals survived and reproduced for up to 17 days after cutting, control should be carried out at least three weeks before the flowering phase to provide a safe margin preventing further flower or seed development.

## Supplementary Information

Below is the link to the electronic supplementary material.

Supplementary Material 1

Supplementary Material 2

Supplementary Material 3

## Data Availability

The raw data used to perform the statistical analyses are provided in the supplementary material file.
